# Impact of a plant-derived agent on bond strength of self-adhesive resin cement luting of glass-fiber posts

**DOI:** 10.1590/0103-644020256465

**Published:** 2025-09-19

**Authors:** Bárbara de Fátima Barbosa de Freitas, Italo Hudson Tavares Maia, Tainah Oliveira Rifane, Victor Pinheiro Feitosa, Diego Lomonaco Vasconcelos de Oliveira, Diego Martins de Paula

**Affiliations:** 1 Paulo Picanço School of Dentistry, Ceará, Brazil.; 2 Department of Operative Dentistry, University of Iowa, College of Dentistry, Iowa City, IA, USA; 3 Department of Chemistry, Federal University of Ceará, Brazil

**Keywords:** Lignin, dentin, self-adhesive cement, push-out bond strength, glass-fiber posts

## Abstract

This study aimed to evaluate the effectiveness of plant-derived compounds, lignin (LIG) and proanthocyanidin (PAC), either alone or in combination with EDTA, as pre-treatments for intraradicular dentin to enhance the push-out bond strength (PBS) of glass fiber posts (GFP) cemented with self-adhesive resin cement. Forty-two healthy human single-rooted premolars were prepared for GFP cementation and divided into the following groups based on the dentin pre-treatment: 1) Control: no pre-treatment; 2) EDTA: 17% EDTA; 3) LIG: 2% LIG; 4) PAC: 2% PAC; 5) EDTA-LIG: 17% EDTA + 2% LIG; and 6) EDTA-PAC: 17% EDTA + 2% PAC. All solutions were applied for 60 seconds. The GFPs were subsequently cemented using the self-adhesive resin cement RelyX U200. The roots (n = 7 per group) were sectioned into 1-mm thick discs and subjected to PBS testing after 1 week and 6 months. Statistical analysis was performed using two-way ANOVA followed by Tukey’s test (p < 0.05). After one week, the Control and EDTA-LIG groups (p = 0.092) exhibited similar and high bond strength values compared to the EDTA, LIG, and EDTA-PAC, while the PAC showed the lowest PBS. After six months, the EDTA-LIG maintained its bond strength values (p < 0.001), with no significant differences from the other groups, except for the PAC. In conclusion, the intraradicular dentin pre-treatments exhibited behavior comparable to untreated RelyX U200 cement in the bonding between the fiberglass post and intraradicular dentin, except proanthocyanidin.

## Resumo

O objetivo deste estudo foi avaliar a eficácia dos compostos derivados de plantas, lignina (LIG) e proantocianidina (PAC), isolados ou associados ao EDTA, como pré-tratamentos para a dentina intraradicular, com o intuito de melhorar a resistência de união push-out (PBS) de pinos de fibra de vidro (GFP) cimentados com cimento resinoso autoadesivo. Quarenta e dois pré-molares humanos, saudáveis e com raiz única, foram preparados para a cimentação dos GFPs e divididos nos seguintes grupos de acordo com o pré-tratamento da dentina: 1) Controle: sem pré-tratamento; 2) EDTA: 17% EDTA; 3) LIG: 2% LIG; 4) PAC: 2% PAC; 5) EDTA-LIG: 17% EDTA + 2% LIG; e 6) EDTA-PAC: 17% EDTA + 2% PAC. Todas as soluções foram aplicadas por 60 segundos. Os GFPs foram então cimentados utilizando o cimento resinoso autoadesivo RelyX U200. As raízes (n = 7 por grupo) foram seccionadas em discos de 1 mm de espessura e submetidas ao teste de PBS após 1 semana e 6 meses. A análise estatística foi realizada utilizando ANOVA de dois fatores, seguida pelo teste de Tukey (p < 0,05). Após uma semana, os grupos Controle e EDTA-LIG (p = 0,092) apresentaram valores de resistência de união similares e elevados aos dos grupos EDTA, LIG e EDTA-PAC, enquanto o grupo PAC exibiu os menores valores de resistência. Após seis meses, o grupo EDTA-LIG manteve os maiores valores de resistência de união (p < 0,001), sem diferenças significativas em relação aos grupos Controle, EDTA, LIG e EDTA-PAC. Em conclusão, os pré-tratamentos da dentina intraradicular apresentaram comportamento comparável ao cimento RelyX U200 não tratado na adesão entre o pino de fibra de vidro e a dentina intraradicular, com exceção da proantocianidina.

**Figure:**
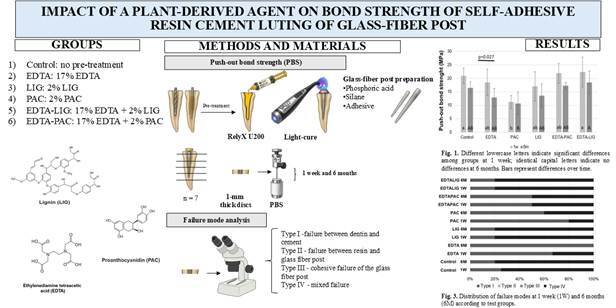

## Introduction

The cementation of glass fiber posts (GFP) is crucial for full crowns on endodontically treated teeth with limited remaining structure ^1^ ^,^ ^2^. GFPs are widely employed to support the retention of prosthetic restorations due to their aesthetic, ease of removal, reduced risk of corrosion, and modulus of elasticity comparable to that of root dentin. Additionally, GFPs enhance stress distribution between the tooth remnant and the cementation interface ^3^ ^,^ ^4^.

GFPs are cemented with dual-cure resin cement, classified as conventional or self-adhesive resin cement. Conventional resin cements require acid etching of the dentin with phosphoric acid, while self-adhesive resin cements enable prior treatment ^5^ ^,^ ^6^. The use of self-adhesive resin cement simplifies the GFP luting procedure. Acidic monomers that demineralize and infiltrate the dentin, forming chemical bonds with the tissue ^3^ ^,^ ^7^. However, several factors affect dentin bonding, including contamination, the smear layer's presence, and the root dentin's morphology and composition. These factors may prevent the penetration of self-adhesive resin cement and compromise the durability of the restoration ^1^ ^,^ ^2^ ^,^ ^3^ ^,^ ^4^.

Therefore, to mitigate the aforementioned effects, various methods and alternatives are being explored, including the use of less acidic conditioners such as ethylenediaminetetraacetic acid (EDTA), titanium tetrafluoride (TiF₄) ^10^, ethylenediamine tetramethylene phosphonate (EDTF) ^11^, and plant-derived agents. EDTA is employed for acid etching of root dentin. However, it exhibits low wetting capacity and the cement's high viscosity, which may adversely impact its performance ^12^ ^,^ ^13^.

Plant-derived/natural agents for dentin have been mentioned in the literature as potential alternatives to improve intraradicular dentin bonding, for example, cardanol and cardol ^14^. These extracts rich in polyphenols have shown effective bioactivity, increased biomechanical properties of collagen, and increased cross-linking density, which makes them promising for this purpose ^15^. Extracts from grape seeds (*Vitis vinifera*) and leaves of the green tea plant (*Camellia sinensis*), among others ^16^ ^,^ ^15^. Previous research demonstrates that using proanthocyanidin (PAC) as a dentin pre-treatment improved the mechanical properties of collagen and reduced the biodegradation rates ^17^. PACs may exhibit limited penetration into demineralized dentin due to their high molecular weight, which restricts their mobility through the narrow dentinal tubules.

Additionally, larger molecules with greater volume and more complex structures face challenges in diffusing through the dense collagen fiber network within the dentin matrix. PACs typically have a molecular weight ranging from 500 to 3,000 daltons. In contrast, the diameter of dentinal tubules generally ranges from 1 to 4 micrometers (µm) in superficial layers, potentially increasing in deeper regions or following demineralization treatments, further hindering the penetration of these molecules.

To overcome these limitations, an interesting alternative is lignin, a natural polymer of plant origin derived from wood combustion in the paper industry. Due to its chemical composition rich in polyphenols, lignin demonstrates significant potential to increase the functional stability of the dentin organic matrix, achieve bond strength, and mitigate collagen degradation ^19^ ^,^ ^20^. Lignin has been employed as a pre-treatment agent for coronal dentin, incorporated into phosphoric acid to enhance cross-linking with dentin without compromising the degree of conversion and reducing nanoleakage ^19^ ^,^ ^20^. Due to its structure rich in phenolic groups, lignin can chemically interact with dentin proteins, forming covalent bonds with amine groups (-NH₂), amide groups (-CONH-), as well as amino acids such as glycine and proline. While promising results have been observed in coronal dentin, the potential of such biomodification agents for root dentin bonding remains to be further explored.

This study aimed to evaluate the influence of lignin and proanthocyanidin on the push-out bond strength (PBS) of glass fiber posts cemented with self-adhesive resin cement. The null hypothesis was that 1) there would be no difference between the pre-treatments in terms of PBS, and 2) there would be no difference in PBS values between 1 week and 6 months.

## Material and methods

### Preparation of Dentin Biomodification Solutions

**Figure 1:**
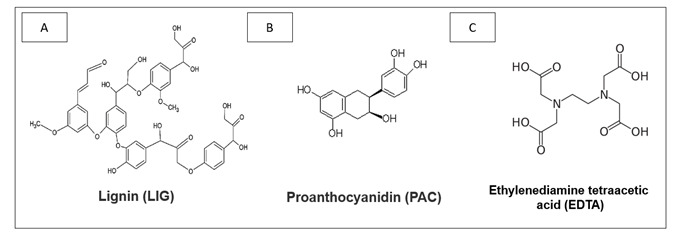
Molecular structures of the components of intraradicular dentin pre-treatments: **A.** LIG **B.** PAC **C.** EDTA.

The solutions were prepared according to the De Paula et al. 2022 ^19^ protocol with some modifications. The lignin (LIG) used in the study was sourced from Eucalyptus plants, generously provided by Suzano Papel e Celulose (Suzano, Fortaleza, Brazil). LIG was diluted in water/ethanol (1:1) to achieve a concentration of 2% by weight. Proanthocyanidin (PAC) is extracted from grape seeds of *Vitis vinifera* and obtained from the company Meganatural (Gold, Madera, CA, USA). PAC was dissolved in water/ethanol (1:1) at a concentration of 2% by weight ^19^. The solution was stirred for 5 minutes at 25°C and double-filtered (19, 20). The EDTA used in the study was obtained from Sigma Aldrich (St. Louis, MO, USA), and the solution was used at a concentration of 17%. The groups were divided according to the pre-treatment solutions, as described in Table 1, and the molecular structure of each component is shown in Figure 1.

**Table 1:** Division of groups according to intraradicular dentin pre-treatment.

Groups	Composition (pre-treament)
Control	No pre-treatment
EDTA	17% EDTA, distilled water
LIG	2% lignin, distilled water and ethanol
PAC	2% proanthocyanidin, distilled water and ethanol
EDTA-LIG	17% EDTA, 2% lignin, distilled water and ethanol
EDTA-PAC	17% EDTA, 2% proanthocyanidin, distilled water and ethanol

### Tooth Preparation and Bonding Procedure

A total of 42 healthy human single-rooted premolar teeth were utilized in this study, following approval from the ethics committee (CAAE: 83249618.0.0000.5054). The teeth were preserved in 0.01% thymol for two months and refrigerated. The teeth (n=7 per group) were sectioned below the cementoenamel junction, perpendicular to the longitudinal axis, using a water-cooled diamond disc in a metallographic cutting machine (Isomet 4000; Buehler, Lake Bluff, USA). The root canal was enlarged using #15, #20, #25, and #30 files (Dentsply Maillefer, Ballaigues, Switzerland), followed by #1, #2, and #3 large drills (Dentsply Maillefer, Ballaigues, Switzerland), as detailed by Cecchin et al. ^21^.

After each use of the files, the root canals were irrigated with 5 mL of 0.9% sodium chloride (NaCl) solution to remove debris from the instrumentation. The apical region (1 mm) was left uninstrumented to prevent solutions and resin cement extrusion. Following root canal preparation, the teeth were treated with the experimental solutions for 60 seconds (Control, EDTA, LIG, PAC, EDTA-LIG, EDTA-PAC), followed by rinsing and drying with absorbent paper points **(**MK Life, São Pulo, Brazil). No pre-treatment was applied to the root dentin in the negative Control group.

The GFPs were treated with 37% phosphoric acid (FGM, Joinville, SC, Brazil) for surface conditioning, then silanized (Dentsply, York, PA, USA) for 20 seconds and dried with compressed air for 1 minute. The resin cement was injected into the root canal using a Centrix EC Speed Slot syringe with a 20-G needle. The GFPs were placed into the root canal and held under digital pressure for 20 seconds. The intracanal glass fiber posts (GFPs), No. 1 posts (WhitePost, FGM, Joinville, SC, Brazil), were cemented using RelyX U200 dual-cure self-adhesive resin cement (3M ESPE, St. Paul, MN, USA). The teeth were not subjected to endodontic treatment. The self-adhesive resin cement was applied directly to the root dentin, and GFP was inserted and light-cured for 40 seconds on each aspect (mesial, distal, buccal, and lingual/palatal) using Valo (South Jordan, UT, USA).

### Push-out bond strength test (PBS)

Seven days after the cementation of the GFP in distilled water, the samples were sectioned horizontally using a diamond disk under continuous water cooling in a metallographic cutting machine (Isomet 4000; Buehler, Lake Bluff, US). Six to seven 1 mm thick slabs were obtained per tooth, excluding the sample closest to the cementoenamel junction. Half of the samples were stored for 1 week, while the remaining samples were stored for 6 months in distilled water at 37°C before testing.

The PBS test used a universal testing machine (EMIC INSTRON 23 - 2S, EMIC). A plunger with a 1.0 mm diameter tip was adapted to the testing machine and positioned without touching the root wall. The load was applied from the surface to the cervical apically. The test was performed on a universal testing machine (EMIC INSTRON 23 - 2S, EMIC) with a 2000 N load cell (CCE 2000N; EMIC) at a 0.5 mm/min speed. The maximum extrusion load in N was expressed in megapascals (MPa), and the failure load was recorded in newtons (N) and divided by the area (mm²) of the post-dentin interface. The area calculation was performed using the formula π (R + r)[h² + (R - r)²], where "R" represents the larger radius, "r" is the smaller radius, and "h" is the thickness of the slice ^22^. PBS data from the same tooth were tabulated, and the means were used as a statistical unit.

### Failure mode

Failure mode analysis was conducted using Stereo Microscopy (OMAX Dual LED 3D) at 20x magnification. The identified failure modes were categorized as follows: Type I indicated failure between dentin and cement, Type II indicated failure between resin and glass fiber post, Type III indicated cohesive failure of the glass fiber post, and Type IV represented mixed failure involving a combination of two or more failure types.

### Statistical analysis

The PBS data was analyzed for normal distribution and homoscedasticity using the Shapiro-Wilk and Levene tests. Subsequently, the data was analyzed by two-way ANOVA (factors: "surface treatment" and "evaluation time") and Tukey post-hoc (α=0.05). The bond strength was statistically analyzed using SigmaStat software (Systat, Palo Alto, CA, USA).

## Results

### Push-out Bond Strength Test (PBS) and Failure Mode

**Figure 2:**
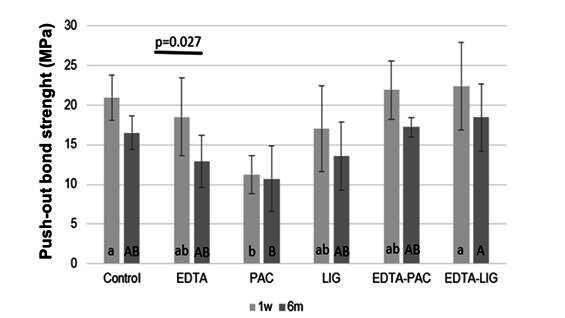
Push-out bond strength outcomes (means and standard deviations) in MPa. Different lowercase letters highlight significant differences among groups at 1 week. Similar capital letters indicate no statistical difference among groups in 6 months. The bars above columns depict the p-value for significant differences between time evaluations.

The results of the push-out bond strength test are shown in Figure 2. Storage time significantly influenced the PBS values in the EDTA group (p = 0.027). After one week, the Control and EDTA-LIG groups (p = 0.092) demonstrated statistically similar bond strength values to the EDTA, LIG, and EDTA-PAC groups, which exhibited the highest values. In contrast, the P, AC group showed the lowest average bond strength. After six months, the EDTA-LIG group maintained the highest bond strength values (p < 0.001), while intermediate values were observed in the Control, EDTA, LIG, and EDTA-PAC groups.

**Figure 3:**
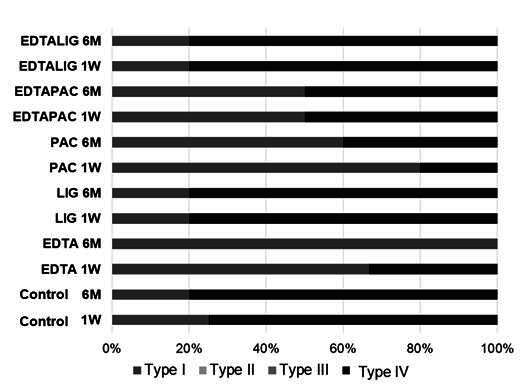
Distribution of failure modes at 1 week (1W) and 6 months (6M) according to test groups. Type I: failure between dentin and cement; Type II: failure between resin and glass fiber post; Type III: cohesive failure of the glass fiber post; and Type IV: mixed failure (a combination of two or more failure types).

Figure 3 illustrates the distribution of failure modes. The Control, LIG, EDTA-LIG, and EDTA-PAC groups predominantly displayed mixed failures (type IV). In contrast, the PAC and EDTA groups exhibited more adhesive failures (type I) at both time points.

## Discussion

Based on the results, the combination of PAC/LIG and EDTA yielded outcomes similar to those of the Control group, differing from the performance of each natural biomodification agent used alone. This suggests that various pre-treatments have distinct effects on the intraradicular bonding of fiberglass posts cemented with self-adhesive resin cement. Consequently, the null hypothesis was rejected, as the dentin pre-treatments demonstrated varied performances. Furthermore, the second hypothesis was also rejected, as EDTA showed a difference in PBS values at 1 week and after 6 months.

PAC was utilized as a natural dentin biomodification agent to compare with lignin. This natural agent, a phenolic compound prevalent in woody and herbaceous plants (15-20), is classified as a condensed tannin. It features highly hydroxylated structures capable of forming insoluble complexes with carbohydrates and proteins ^15^ ^,^ ^16^ ^,^ ^17^ ^,^ ^18^ ^,^ ^19^ ^,^ ^20^. The complexes formed between PAC and the collagen matrix are stabilized primarily by hydrogen bonds between the protein's amide carbonyl and the phenolic hydroxyl groups, in addition to covalent and hydrophobic bonds ^15^ ^,^ ^16^ ^,^ ^17^ ^,^ ^18^ ^,^ ^19^ ^,^ ^20^. In this study, the application time was standardized to 60 seconds, as Moreira et al. ^23^ recommended, to ensure clinical applicability and the PAC concentration was set at 2% (18).

Additionally, PAC enhances collagen cross-linking, inhibits bacterial proteases and matrix metalloproteinases (MMPs), and regulates inflammatory mediators associated with periodontitis ^15^ ^,^ ^16^ ^,^ ^17^ ^,^ ^18^. The effectiveness of collagen matrix cross-linking depends on the number of hydroxyl groups and the molecular structure of PACs, which includes aromatic rings that facilitate hydrophobic bonds ^15^ ^,^ ^16^ ^,^ ^17^ ^,^ ^18^ ^,^ ^19^. However, PAC's high molecular weight and size limit its deep penetration into the collagen matrix, leading to lower PBS values and increased adhesive failures. These values remained consistent after aging, with bond stability attributed to cross-linked dentin.

Combining EDTA with PAC resulted in higher bond strength outcomes than PAC alone. This improvement can be attributed to the weakly acidic nature of EDTA, a chelating agent used to demineralize dentin. EDTA effectively removes the smear layer on the canal wall surface and within the dentinal tubules formed during endodontic treatment, enhancing the hybrid layer ^18^ ^,^ ^20^. Although EDTA may not remove the smear layer as effectively as stronger acids like phosphoric acid, it facilitates the infiltration of resin cement into dentinal tubules and collagen fibers, enhancing bond strength through mechanical interlocking ^12^ ^,^ ^13^. However, this mechanism alone is insufficient to ensure long-term restoration durability. Therefore, natural agents such as proanthocyanidins (PAC) and lignin (LIG) are necessary. EDTA is a chelating agent, primarily demineralizing dentin by removing calcium ions from the dentin matrix. This process exposes collagen fibers, making them more accessible for penetration by other substances and increasing the surface area for interaction with PAC. This mechanism can enhance the stability of collagen fibers, making them more resistant to enzymatic degradation, particularly by matrix metalloproteinases (MMPs), which could compromise the durability of the bond.

As previously discussed, conditioning the dentin surface with EDTA enhances the interaction between PAC and dentin ^12^ ^,^ ^13^. PAC binds to the collagen structure through covalent and hydrogen bonds, interacting with the amine groups of lysine and hydroxylysine and carboxyl groups of aspartic and glutamic acids ^16^ ^,^ ^17^ ^,^ ^18^. This interaction improves cross-linking and increases the elastic modulus of collagen while protecting against degradation, as evidenced by the preservation of bond strength values over time (EDTA-PAC).

The use of natural products to enhance dentin bonding is an emerging approach. Renewable natural products, such as lignin, can be sourced as industrial by-products from processes like papermaking ^18^ ^,^ ^19^ ^,^ ^20^. Lignin has a hydrophobic chemical structure (Figure 1, 1A), characterized by numerous aromatic rings. Its stability arises from the structural complexity generated by multiple bonding possibilities among the p-hydroxyphenyl, guaiacol, and syringyl subunits ^19^ ^,^ ^20^. This structural complexity influences lignin's resistance to the acidity of EDTA solutions.

Moreover, lignin's highly branched and amorphous molecular architecture, with numerous hydroxyl groups, enables it to form three-dimensional cross-links with collagen effectively, providing protection when applied for 60 seconds ^19^ ^,^ ^20^. The molecular size of lignin does not impede collagen penetration, as it consists of a heteropolymer aromatic complex with three main units (Figure 1, 1A), characterized by hydroxyl and methoxyl functionalities. The selection of a 2% concentration, based on previous research, does not compromise the adhesive's degree of conversion, effectively restores the modulus of elasticity of demineralized dentin, maintains dentin bond strength values, and results in mixed failures ^19^ ^,^ ^20^.

The U200 group did not show significant differences in bond strength values after aging. This behavior can be attributed to the mild and superficial demineralization of dentin promoted by the 10-Methacryloyloxydecyl Dihydrogen Phosphate (10-MDP) monomer in the cement, which exposes collagen fibrils and allows for subsequent infiltration by resin monomers ^24^. Additionally, the initial acidic pH of the cement, which facilitates demineralization, is neutralized during the reaction with filler particles and dental structure, reducing the risk of water absorption and the consequent degradation of polymer properties ^25^. Therefore, the addition of the EDTA-LIG pre-treatment did not demonstrate a statistically significant difference compared to the Control group after 6 months, indicating the need for further investigation to justify the inclusion of an additional clinical step.

Therefore, the pre-treatment with EDTA and LIG resulted in stable bond strength to intraradicular dentin, even after water aging. However, the study has limitations, including the lack of evaluation of chemical characteristics such as the degree of conversion in different root thirds, characterization of collagen cross-linking, and analysis of bond strength over extended periods. Future research should explore the use of these biomodifiers in the cementation of fiberglass posts with conventional resin cement or in combination with universal cement.

## Conclusion

The intraradicular dentin pre-treatments exhibited behavior similar to self-adhesive cement in bonding between the fiberglass post and intraradicular dentin, except for proanthocyanidin.
